# Expression Level of the *DREB2*-Type Gene, Identified with Amplifluor SNP Markers, Correlates with Performance, and Tolerance to Dehydration in Bread Wheat Cultivars from Northern Kazakhstan

**DOI:** 10.3389/fpls.2016.01736

**Published:** 2016-11-18

**Authors:** Yuri Shavrukov, Aibek Zhumalin, Dauren Serikbay, Makpal Botayeva, Ainur Otemisova, Aiman Absattarova, Grigoriy Sereda, Sergey Sereda, Vladimir Shvidchenko, Arysgul Turbekova, Satyvaldy Jatayev, Sergiy Lopato, Kathleen Soole, Peter Langridge

**Affiliations:** ^1^School of Agriculture, Food and Wine, Faculty of Sciences, University of AdelaideUrrbrae, SA, Australia; ^2^School of Biological Sciences, Flinders University, Bedford ParkSA, Australia; ^3^Faculty of Agronomy, S. Seifullin Kazakh AgroTechnical UniversityAstana Kazakhstan; ^4^National Agricultural Research Education CentreAstana, Kazakhstan; ^5^Karaganda Research Institute of Plant Industry and BreedingKaraganda, Kazakhstan

**Keywords:** Amplifluor SNP, bread wheat, dehydration, *DREB2*, gene expression, genotyping, grain yield

## Abstract

A panel of 89 local commercial cultivars of bread wheat was tested in field trials in the dry conditions of Northern Kazakhstan. Two distinct groups of cultivars (six cultivars in each group), which had the highest and the lowest grain yield under drought were selected for further experiments. A dehydration test conducted on detached leaves indicated a strong association between rates of water loss in plants from the first group with highest grain yield production in the dry environment relative to the second group. Modern high-throughput Amplifluor Single Nucleotide Polymorphism (SNP) technology was applied to study allelic variations in a series of drought-responsive genes using 19 SNP markers. Genotyping of an SNP in the *TaDREB5* (*DREB2*-type) gene using the Amplifluor SNP marker KATU48 revealed clear allele distribution across the entire panel of wheat accessions, and distinguished between the two groups of cultivars with high and low yield under drought. Significant differences in expression levels of *TaDREB5* were revealed by qRT-PCR. Most wheat plants from the first group of cultivars with high grain yield showed slight up-regulation in the TaDREB5 transcript in dehydrated leaves. In contrast, expression of *TaDREB5* in plants from the second group of cultivars with low grain yield was significantly down-regulated. It was found that SNPs did not alter the amino acid sequence of TaDREB5 protein. Thus, a possible explanation is that alternative splicing and up-stream regulation of *TaDREB5* may be affected by SNP, but these hypotheses require additional analysis (and will be the focus of future studies).

## Introduction

Drought is a major cause of crop losses worldwide. It is one of the main restrictions on wheat production, and is predicted to become even more serious as a result of global climate changes (Ashraf, 2010; Langridge and Reynolds, 2015). Dehydration of plant tissues is a component of drought stress and plants protect themselves from dehydration at the molecular, cellular, tissue, and whole plant levels (Bohnert and Jensen, 1996; Kosová et al., 2014). The main change that occurs at the molecular level is a re-arrangement in transcriptomes, which includes either the switching off or reduction in activity of genes responsible for plant growth and development; and the induction of large numbers of stress-responsive genes.

Among regulatory proteins, the most important role belongs to Transcription Factors (TFs). Genome-wide identification of TF genes has revealed that around 20% of genes in plant genomes encode TFs (Iida et al., 2005; Xiong et al., 2005). Most TF gene families contain stress-responsive and stress-activated members. Among the main and best-characterized drought-responsive TFs is a subfamily of Drought Responsive Element Binding (DREB), which comprise one of two subfamilies of the APETALA2/Ethylene Responsive Element Binding Factor (AP2/ERF) family of TFs containing a single AP2 domain (Agarwal et al., 2006; Lata and Prasad, 2011; Mizoi et al., 2013; Rehman and Mahmood, 2015). Most DREB TFs specifically recognize the dehydration-responsive element/C-repeat (DRE/CRT), which was initially identified in the promoters of drought- and cold-responsive genes (Yamaguchi-Shinozaki and Shinozaki, 1994; Stockinger et al., 1997).

Drought Responsive Element Binding TFs were divided into six groups (A-1–A-6) based on the presence of characteristic conserved protein sequences, domains and motifs, and their ability to be activated in particular stress response pathways (Sakuma et al., 2002; Nakano et al., 2006). Subgroup A-1 consists of cold and dehydration-responsive TFs (DREB1/CBF-type); whereas A-2 contains salt, dehydration, and heat-responsive TFs (DREB2-type). The expression and consequent activity of the *DREB1*/*CBF* genes is regulated at the transcriptional level and the activity of the *DREB2*-type genes is controlled at transcriptional, post-transcriptional and post-translational levels. Alternative splicing has been suggested for DREB2-type TFs from grasses as a possible post-transcriptional mechanism of activity regulation (Egawa et al., 2006; Qin et al., 2007; Matsukura et al., 2010; Vainonen et al., 2012). For example, in wheat three alternative transcripts of the *Wdreb2* gene can be produced through alternative splicing. Under drought and high salinity, the amount of the correctly spliced form increases, while low temperatures increase the amount of all three forms (Egawa et al., 2006). At the post-translational level, abundance, and activity of DREB2-type TFs is controlled by protein phosphorylation and ubiquitin-mediated degradation (Liu et al., 1998; Agarwal et al., 2007). In most reported cases constitutive overexpression of *DREB* genes including *DREB*2-type TFs led to a negative influence of the transgene on plant development (Shen et al., 2003; Qin et al., 2007; Kobayashi et al., 2008; Morran et al., 2011). No yield improvement under drought has been reported so far for transgenic plants with overexpressed *DREB2*-type genes.

Single Nucleotide Polymorphism (SNP) molecular markers are widely used, especially in bread wheat where the hexaploid genome results in a higher SNP rate than most other crops. However, the probability of a nucleotide substitution occurring in an individual genome of bread wheat in isolation is much smaller compared to many other species (Shavrukov, 2016). Different technologies of SNP detection have been developed for wheat to study various aspects of genetic polymorphism in cultivars, breeding lines and mapping populations, utilizing both conventional and transgenic breeding approaches. Amplifluor (Amplification with Fluorescence) is a recent method based on a similar technology platform for SNP analysis as KASP (Kompetitive Allele Specific PCR) (Saxena et al., 2012; Khera et al., 2013; He et al., 2014; Yuan et al., 2014). Ampliflour SNP markers have a simple design, with universal probes, fluorescent labels and primers; and with its low operational costs it can be easily adapted for high-throughput methods.

Despite its obvious advantages, Amplifluor SNP markers are rarely used in plant biology although they have been applied in the model species *Arabidopsis thaliana* (Giancola et al., 2006). No published information using Amplifluor SNP exists for cereal crops, including bread wheat. Therefore, our current study can be considered a pilot project in the application of Amplifluor to assay SNPs in bread wheat cultivars varying in their tolerance or sensitivity to the drought conditions in Northern Kazakhstan.

The aims of this study were to: (1) select candidate drought-responsive genes from an SNP database, (2) identify candidate or marker genes responsible for drought tolerance and performance in dry conditions using newly developed Amplifluor SNP markers and wheat cultivars varying in tolerance or sensitivity to the drought environment of Northern Kazakhstan, and (3) to analyse the expression levels of the identified *DREB2*-type TF, *TaDREB5*, by qRT-PCR in dehydrated and control leaves from plants in a panel of selected Kazakh wheat cultivars.

## Materials and Methods

### Plant Material and Drought Score in Field Trials

An initial panel of 89 local wheat cultivars were analyzed for grain yield in field trials with 12 plots each year, 4 rows × 10 m in each plot, over 3 years from 2013 to 2015 in the Karaganda region of Northern Kazakhstan (**Supplementary Table S1**). This area is characterized as a very dry environment for plant growth and total rainfall varied between 107 mm and 171 mm during the vegetative growth period with average temperature 26–28°C. Seeds of all 89 wheat cultivars were harvested and plants were grown in the field under well-watered conditions for leaf sampling and molecular experiments in close vicinity to the laboratory in Astana in 2016.

### Dehydration and Water Loss in Detached Leaves

Five whole flag leaves at anthesis were collected from five randomly selected plants of each cultivar and sealed in plastic bags. Fresh weights of detached leaves were measured on a scale (Shimadzu, Japan) accurate to four-decimal places in the laboratory. The same leaves were then dehydrated on the bench at room temperature (about 22°C) for 6 h, until wilting was clearly observed. Weights of dehydrated leaves were measured and the ratio of water loss was calculated in each leaf using the formula: (WFL – WDL)/WFL, where WFL is Weight of fresh leaf and WDL is Weight of dehydrated leaf, both in grams.

### DNA Extraction and Amplifluor SNP Analysis

Single leaves were combined from five randomly selected plants in each cultivar at the tillering stage, collected in 10-ml plastic tubes and frozen at -80°C prior to DNA extraction. Leaf samples were transferred from liquid nitrogen and ground with two 9-mm stainless ball bearings. A phenol-chloroform method of total DNA extraction was used as described earlier (Sharp et al., 1988), and the quality of isolated DNA was checked by PCR.

Amplifluor SNP analysis was based on the principals of the published information (Myakishev et al., 2001; Rickert et al., 2004; Giancola et al., 2006; Khripin, 2006; Hamilton et al., 2010; Löfström et al., 2015) and was carried out using a QuantStudio-7 Real-Time PCR instrument (ThermoFisher Scientific, USA). Two universal probes with either a FAM or VIC fluorescent label on the 5′-end and BHQ1 (Black Hole Quencher 1) in the middle were synthesized by DNA Synthesis Company (Moscow, Russia). Three gene-specific primers (two forward and one common reverse) were synthesized by Biosan Company (Novosibirsk, Russia). Each of the probes has a unique tail on the 3′-end, and this tail is identical to those on the 5′-end of each gene-specific forward primers. The last nucleotide on the 3′-end of the gene-specific forward primer is designed to match with an SNP. In fact, two gene-specific forward primers have identical sequences in the middle but different tails on the 5′-end and differ by a single nucleotide on the 3′-end. Therefore, amplification of a PCR product with either first or second gene-specific forward primers is dependent on alleles of the SNP. Amplification using the fluorescent probe is carried out and wavelength-specific absorption is accurately determined and discriminated by the qPCR machine. The protocol for Amplifluor SNP genotyping using any qPCR instrument or PCR thermal cycler was based on those reported earlier (Rickert et al., 2004; Giancola et al., 2006; Khripin, 2006) with small modifications; amplification was carried out in either 96- or 384-well microplates using a 10 μl or 5 μl total reaction volume for PCR, respectively.

The PCR cocktail in each well contained 2x Master-Mix with the following reagents in final concentrations: 1xPCR Buffer, 1.8 mM MgCl_2_, 0.25 μM each fluorescent label probe, 0.2 mM each of dNTPs, 0.15 mM of each forward primer, 0.78 mM of reverse primer and 0.5 units of Taq DNA polymerase (GenLab, Astana, Kazakhstan). Half of the PCR volume was genomic DNA, adjusted for 10 ng/μl, and 5.0 μl or 2.5 μl of each DNA sample was added in 96- or 384-well microplates, respectively. One micro liter of 1:100 diluted Low ROX was added as a passive Reference label to the Master-Mix as prescribed for the qPCR instrument. Digital single- and multichannel pipette dispensers (Eppendorf, Germany) were used for loading Master-Mix and DNA samples, respectively, using cold-block to avoid evaporation and with accurate manual loading (without a robotic system).

PCR was run following a program adjusted from those published earlier (Rickert et al., 2004; Khripin, 2006) and included initial denaturation for 95°C for 1 min; 50 cycles of 95°C for 30 s, 50°for 30 s, and 72°C for 50 s; and final extension for 72°C for 5 min. Genotyping with SNP calling was determined automatically by software accompanying the instrument, but each SNP result was checked manually using amplification curves and final allele discrimination. Experiments were repeated twice over different days, where technical replicates confirmed the confidence of SNP calls.

### SNP Selection for Potential Candidates in Drought-Responsive Genes

Nineteen SNPs were selected from the publicly available database Cereal DB^1^ with supported BLAST results for drought-responsive candidate genes. Some of the genes have similar or identical annotations, but SNP were located in different regions of the gene. A list of the 19 Amplifluor SNP markers, sequences with SNP positions, references to the annotated contigs and corresponding BLAST results are provided in the **Supplementary Table S2**.

### RNA Extraction, cDNA Construction and qPCR Analysis

Three plants were randomly selected from each cultivar at the start of anthesis and three flag leaves were collected individually into 10-ml plastic tubes. Three tubes with three flag leaves from independent plants in each cultivar were frozen immediately in liquid nitrogen, transported to the laboratory and stored at -80°C until RNA extraction. These leaf samples were designated as controls. Identical leaf samples from the same plants were collected, transported to the laboratory and left to dry on the bench for 6 h at room temperature as described above to facilitate water loss from the dehydrated leaves. Dehydrated leaf samples were returned to the plastic tubes and frozen together with control samples at -80°C until RNA extraction. Frozen leaf samples were ground as described above for DNA extraction. TRIsol-like reagent was used for RNA extraction following the protocol published earlier (Shavrukov et al., 2013) and the quality of RNA was checked on agarose gels. After treatment with 1 μl of DNase (Quigen, Germany), the MoMLV Reverse Transcriptase kit (Biosan, Novosibirsk, Russia) was used to construct first-strand cDNA reactions which included 2 μg of each RNA sample, oligo(dT)_20_ primer and dNTPs as recommended by the manufacturer. All cDNA samples were checked for quality using PCR and yielded the expected bands on agarose gels.

Diluted (1:10) cDNA samples were used for qPCR analyses in the same instrument as mentioned above for SNP genotyping, a QuantStudio-7 Real-Time PCR (ThermoFisher Scientific, USA). The total volume of 10 μl qPCR reactions included 5 μl of 2xKAPA SYBR FAST (KAPA Biosystems, USA), 4 μl of diluted cDNA, and 1 μl of mixed three gene-specific primers, the same as used above for the SNP genotyping (1.5 μM of each forward primers and 3 μM of common reverse primer). Expression data for the target gene were normalized using the average expression of two housekeeping genes, for Ta30768, Glyceraldehyde-3-phosphate dehydrogenase (*GAPDH)* and for Ta2291, ADP-ribosylation factor (*ADPRF*) (Paolacci et al., 2009).

### Statistical Analysis

Average and standard errors were calculated using standard Excel software. Least significant difference LSD_(0.05)_ was calculated using ANOVA. Probabilities for significance were calculated using Student’s *t*-test. Fisher’s exact test of independence was used for analysis of Amplifluor SNP marker distribution in the studied cultivars, and probability was calculated^2^. The null-hypothesis supposes that data for allele distribution in the two groups of cultivars do not differ in each pair-group representing the same distribution. The null-hypothesis can be rejected if the calculated probability in the Fisher’s exact test of independence is less than *p* < 0.05 or *p* < 0.01, indicating that there is significant or highly significant differences in allele distributions in each pair-groups.

## Results

### Analysis of Seed Production in Dry Conditions and Water Loss in Dehydrated Plants of Selected Cultivars of Bread Wheat in Northern Kazakhstan

An initial panel of 89 wheat cultivars were evaluated for grain yield in field trials over three consecutive years (2013–2015) in the Karaganda region of Kazakhstan (**Supplementary Table S1**). Six cultivars that showed consistently highest yield and six cultivars with lowest yields were selected for further analyses (**Table 1**). Despite the variability, grain yield of each cultivar from the first group was significantly (*p* < 0.95, Student’s *t*-test) higher than the yield of each cultivar from the second group.

**Table 1:** Average grain production (3 years, 12 plots each) and Ratio of water loss was measured in the selected cultivars of bread wheat with highest and lowest grain yield in the dry environment of Northern Kazakhstan.

Wheat cultivars	Average grain yield (g/m^2^), 2013–2015	Ratio of water loss in flag leaf after 6 h of dehydration
**Cultivars with the highest yield**		
Akmola 2	289.5 ± 104.6	0.525 ± 0.020
Aktyubinka	199.8 ± 62.6	0.529 ± 0.019
Albidum 188	274.4 ± 75.9	0.528 ± 0.021
Altayskaya 110	191.0 ± 60.9	0.499 ± 0.025
Karabalykskaya 92	179.9 ± 31.1	0.570 ± 0.013
Saratovskaya 60	214.6 ± 70.7	0.555 ± 0.028
**Cultivars with the lowest yield**		
Astana 2	73.4 ± 19.9	0.613 ± 0.029
Saratovskaya 55	96.8 ± 18.1	0.616 ± 0.048
Vera	96.9 ± 25.9	0.606 ± 0.009
Volgouralskaya	83.3 ± 2.2	0.614 ± 0.015
Yugo-Vostochnaya 2	81.9 ± 9.5	0.591 ± 0.016
Zhenis	94.2 ± 39.1	0.582 ± 0.041

*Ratio of water loss in dehydrated relative to fresh flag leaves was recorded for five plants in each cultivar. Values mean average (*n* = 5) ± SE.*

Relative water loss was studied after 6 h of dehydration using detached flag leaves of the 12 selected cultivars. Results of this experiment (**Table 1**) indicate that leaves of all six cultivars with the highest grain yield lost significantly less water during dehydration compared to the six cultivars with lowest grain yield.

### Amplifluor Analysis of 19 SNPs for Potential Candidates in Drought-Responsive Genes

**FIGURE 1:**
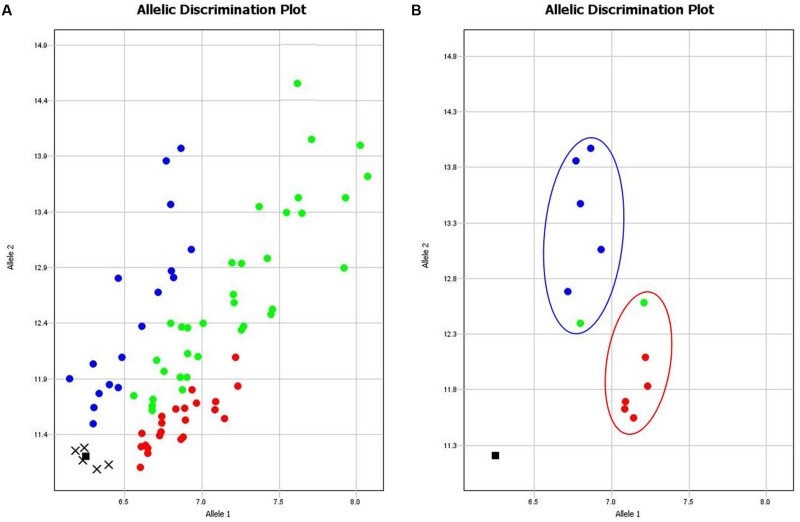
**Results of allelic discrimination of Amplifluor SNP marker KATU48 in 89 bread wheat cultivars from Northern Kazakhstan. (A)** Red and blue dots indicate automatic SNP calls for homozygotes in Allele 1 (*aa*) and Allele 2 (*bb*), respectively, while green dots indicate heterozygotes (*ab*) or mixed genotypes. Undetermined genotypes are represented by crosses and black squares show NTC (No Template Control). Genotyping results were extracted and shown separately **(B)** for two groups of six cultivars each that showed highest (blue oval) and lowest (red oval) grain yield production.

Nineteen Amplifluor SNP markers were analyzed in all 89 cultivars of bread wheat, revealing complex results, where interpretation of genotyping, and phenotyping data required careful analysis. However, SNP analysis in two groups of selected cultivars showed clear contrasts with stable phenotypic characteristics and grain yield. Data presented in **Table 2** indicate a high degree of correlation between Amplifluor SNP marker associations and grain yield in the dry environment. For example, no associations were found in the majority of SNP markers KATU1, 13, 15, 16, 18, 41, and 43–47, where differences in the discriminations did not exceed one cultivar. Markers KATU12, 14, 42, and marker KATU11 showed slight to moderate association, with at least two and three cultivars with different scores, respectively. One marker, KATU40, was monomorphic. The most promising result with the strongest association between the two pools of genotypes was obtained for the single Ampliflour SNP marker KATU48, where all cultivars, except one from each pool, showed clear allele discrimination. The cultivars that did not follow this pattern (Altayskaya 110 and Saratovskaya 55) appeared to be heterozygous or mixed. The KATU48 SNP marker detected a difference in the *TaDREB5* gene, which was subjected to further evaluation of gene expression levels (**Figure 1**).

**Table 2:** Distribution of 19 Amplifluor SNP markers in selected bread wheat cultivars with the highest and lowest grain yield in the dry conditions of Northern Kazakhstan.

Amplifluor SNP marker	Putative gene function	SNP calls in six cultivars with highest yield under drought			SNP calls in six cultivars with lowest yield under drought			Probability for Fisher’s exact test of independence
		*aa*	*bb*	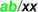	*aa*	*bb*	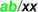
KATU1	Drought-inducible protein 1OS	4	2	0	3	3	0	1.000
KATU11	DRE binding factor 2	0	5	1	3	3	0	0.182
KATU12	Supersensitive to ABA and drought	3	3	0	4	1	1	0.545
KATU13	Supersensitive to ABA and drought	0	5	1	0	5	1	1.000
KATU14	Drought and salt tolerance protein	2	4	0	0	6	0	0.455
KATU15	Drought-induced hydrophobic protein	0	5	1	0	6	0	1.000
KATU16	Drought-inducible protein 1OS	0	6	0	0	5	1	1.000
KATU17	Putative HMG-box with DNA binding protein	0	5	1	3	2	1	0.182
KATU18	Supersensitive to ABA and drought	0	5	1	1	4	1	1.000
KATU39	Drought-induced protein DI	0	4	2	3	3	0	0.091
KATU40	Drought-inducible protein 1OS	0	6	0	0	6	0	1.000
KATU41	EREBP/AP2 type transcription factor	0	4	2	0	5	1	1.000
KATU42	DREB2 transcription factor	2	4	0	0	3	3	0.091
KATU43	Dehydration-responsive element binding protein	2	3	1	3	2	1	1.000
KATU44	AP2 transcriptional activator DRF	1	3	2	1	4	1	1.000
KATU45	Dehydration-responsive factor	2	3	1	2	2	2	1.000
KATU46	EREBP/AP2 type transcription factor	0	6	0	1	5	0	1.000
KATU47	DRE-binding transcription factor	3	3	0	3	2	1	1.000
**KATU48**	DREB2-type transcription factor	0	5	1	5	0	1	**0.004**

*The marker with significant differences (*p* < 0.01) in allele distribution between pair-groups of the cultivars is shown in bold.*

### Expression of *TaDREB5* In Response to Dehydration

**FIGURE 2:**
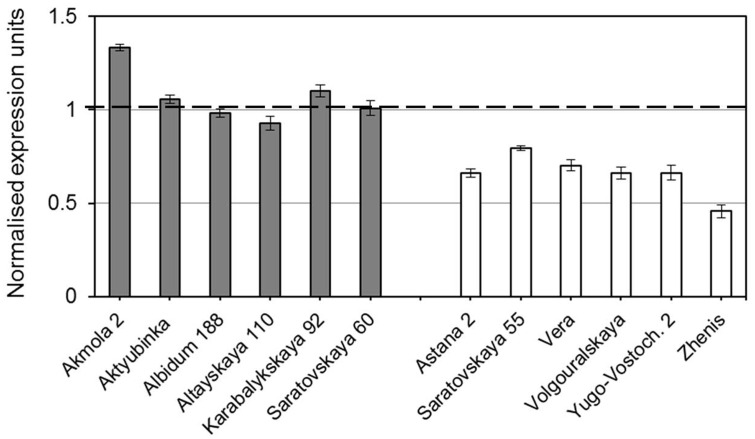
**Normalized expression of the *TaDREB5* gene in dehydrated leaves of plants from two groups of selected cultivars of bread wheat with highest (shaded bars) and lowest (clear bars) grain yield in the dry conditions of Northern Kazakshtan.** Bars represent means for three biological replicates ± Standard errors, the experiment was repeated twice. Expression levels in leaves of control (non-treated) plants for each cultivar were set to one unit and this is indicated by the dashed line. Data were normalized using the average expressions of two housekeeping genes.

Strong differential expression of *TaDREB5* was shown in the experiments using dehydrated leaves of plants from two groups of selected bread wheat cultivars from Northern Kazakhstan (**Figure 2**). In the first group of cultivars with highest grain yield, *TaDREB5* was significantly up-regulated in three out of six cultivars, where Akmola 2 showed 1.3-fold higher gene expression compared to control non-treated leaves from the same plants in each cultivar. No differences in mRNA transcripts of *TaDREB5* were observed in two cultivars while one cultivar showed slightly reduced expression of the gene. In contrast, in all six cultivars with lowest grain yield *TaDREB5* was strongly and significantly (*p* < 0.99, Student’s *t*-test) down-regulated compared to the corresponding control plants. The cultivar Zhenis showed strongest (more than two-fold) down-regulation in dehydrated leaves.

## Discussion

Wheat germplasm in Kazakhstan represent excellent breeding material adapted for strong drought conditions, where terminal drought regularly takes place. In this environment, cultivars with stably high grain yield were strongly pre-selected as drought tolerant. The initial collection of 89 wheat cultivars was studied earlier for genotyping with 90K SNP arrays (Turuspekov et al., 2015). These wheat accessions from Kazakhstan formed a distinct clade on a phylogenetic tree, isolated from others. Our study is based on this published data, and results from our subsequent search for candidate markers and genes among this wheat collection from the dry conditions of Northern Kazakhstan.

Dehydration is an important component of drought stress, and the speed of water loss in detached flag leaves usually correlates with plant tolerance to dehydration (Penuelas et al., 1993; Ergen et al., 2009; Kosová et al., 2014). In our experiments, six selected cultivars of bread wheat with highest grain yield showed a strong association with tolerance to dehydration, while the cultivars most sensitive to dehydration demonstrated the lowest grain yield. Our results confirm those presented by Jäger et al. (2014) which showed that drought sensitive wheat cultivars with less grain production recorded a higher rate of dark-adapted water loss from leaves. The authors claimed that the cuticle thickness of leaves and drought-induced cellular damage play an important role in drought sensitivity in wheat. It was concluded that the decline of relative water content in leaves (in other words, water loss or leaf dehydration) could be a reliable indicator of wheat sensitivity to drought (Jäger et al., 2014).

The application of Amplifluor SNP high-throughput technology facilitates fast and accurate genotyping of wheat material using easily developed markers. Previously, the technique of High Resolution Melting Curve (HRMC) was successfully used to study allelic variation of *TdDREB2* in tetraploid durum wheat (Mondini et al., 2015). In our study, only one Amplifluor SNP marker from a total of 19, KATU48, which identified a SNP in the *DREB2*-type *TaDREB5* gene, showed a strong association with grain yield and tolerance to dehydration. Further genotyping with Amplifluor SNP marker KATU48 will be needed across a wide selection of wheat accessions to be confident that this genetic polymorphism is suitable for broad selection of cultivars adapted to conditions in Northern Kazakhstan and application to breeding programs.

The results presented here show that expression levels of the *TaDREB5* in dehydrated leaves were strongly correlated with dehydration tolerance and grain yield. It was previously demonstrated that *DREB2*-type genes are up-regulated in response to abiotic stresses, involving dehydration, such as drought, high salinity, and extreme temperature changes (Egawa et al., 2006; Qin et al., 2007; Kobayashi et al., 2008; Terashima and Takumi, 2009; Mondini et al., 2015). It was also shown that overexpression of *DREB2*-type genes can enhance tolerance of transgenic plants to several abiotic stresses (Khan, 2011; Bouaziz and Gargouri-Bouzid, 2014). However, correlation between the performance of wheat cultivars under drought in the field and expression levels of *TaDREB5* in dehydrated leaves of the same cultivars is demonstrated here for the first time. Nevertheless, plant response to drought stress involves thousands of genes and the identified *TaDREB5* is just one of several key-genes specific to various wheat genetic backgrounds and different environments.

Further study in a wider range of cultivars is required to verify how the expression level of *TaDREB5* could be correlated with grain yield production under drought. This may also reveal more information regarding the possible intermediate level of *TaDREB5* expression in wheat cultivars from the middle range of yield production in dry conditions. It is known that strong constitutive overexpression of *DREB* genes often causes aberrant development in transgenic plants and penalties in yield. Therefore, based on current results we can conclude that the optimal expression level of *TaDREB5* in wheat plants under drought would be around the level found in the drought tolerant cultivars herein as well as a very modest up-regulation of this level (up to 1.3-fold). In contrast, wheat genotypes with strong down-regulation of *TaDREB5* must be avoided in breeding programs as they are not well adapted to dry environments.

The identified SNP is a substitution of nucleotide A for C in position 687 of the annotated complete mRNA sequence of the wheat *Wdreb2* gene (Acc. AB193608) or in position 849 of the annotated complete mRNA of *TaDREB5A* (Acc. AY781358). These two genes are either close homologs or alternatively spliced forms of the same gene. In both cases the SNP was in the same position in the encoded protein molecules. Further analysis is required to identify differences between the two annotated mRNA and to determine which of them (or both) include the altered SNP. This SNP does not change the amino acid sequence of proteins because the substituted nucleotide is the first nucleotide of Arginine189, which is encoded by both triplets ‘AGA’ and ‘CGA.’ Therefore, this SNP, although an efficient diagnostic tool, is likely to be linked with another change (e.g., recognition sites for an alternative splicing factor), which can affect expression levels and hence the function of *TaDREB5*, a candidate gene for drought (dehydration) tolerance in bread wheat cultivars from Northern Kazakhstan. In contrast, Mondini et al. (2015) reported that SNPs in *TdDREB2* are strongly associated with tolerance to salinity in durum wheat, where the substitution of the single nucleotide caused replacement of Arginine128 for Threonine128 in the TdDREB2 protein.

Changes in the function of *DREB2*-type genes are not always related to the substitution of amino acids in polypeptides. Post-transcriptional alternative splicing is well described for *DREB2*-type genes from different plants (Qin et al., 2007; Zhou et al., 2010) including *WDREB2* from wheat (Egawa et al., 2006; Terashima and Takumi, 2009) and is regulated by different genes. As a detailed analysis of all *TaDREB5* isoforms was not performed in this study, the primers for qRT-PCR were selected so that they would potentially recognize all isoforms and give information about the total number of all alternatively spliced mRNAs of *TaDREB5*. This may point to another mechanism for the up-stream regulation of *TaDREB5* in bread wheat in response to dehydration, which has been proposed previously (Egawa et al., 2006). Down-regulated genes, such as Cold-responsive (*Cor*) and Late embryogenesis abundant (*Lea*) genes, have been reported for *WDREB2* in transgenic tobacco plants (Kobayashi et al., 2008) and will be checked in bread wheat from Northern Kazakhstan in studies to follow.

The role of *DREB2*-type genes in abiotic stress tolerance, especially tolerance to drought and high salinity, and their involvement in two distinct regulatory pathways is well studied in wheat (Egawa et al., 2006) and in maize (Qin et al., 2007). Results of our study show a direct association between levels of the *DREB2*-type *TaDREB5* gene expression in two groups of modern wheat cultivars with contrasting dehydration tolerance and grain yield. The current study could have a significant practical application in wheat breeding programs. It must also be highlighted that the extended study of molecular markers, including further advanced SNP Amplifluor and other marker systems, is required to find more diagnostic markers applicable for Marker Assisted Selection in wheat breeding programs.

## Author Contributions

YS conducted the genotyping experiment and wrote the first version of the manuscript, AZ and DS made qRT-PCR experiments and prepared data, MB and AO carried out Amplifluor SNP analysis, AA coordinated experiments and produced data for SNP analysis, GS, SS, VS, and AT carried out experiments in the field and conducted sampling, SJ coordinated studies and sampling in field and laboratory, SL analyzed *DREB* gene sequences and wrote the corresponding section, KS coordinated the qRT-PCR study and revised the corresponding section, PL supervised the project, revised, and produced the final version of the manuscript.

## Conflict of Interest Statement

The authors declare that the research was conducted in the absence of any commercial or financial relationships that could be construed as a potential conflict of interest.

## Footnotes

1 http://www.cerealsdb.uk.net/cerealgenomics/CerealsDB

2 http://www.biostathandbook.com/fishers.html

## Supplementary Material

TABLE S1 **Average grain production (3 years, 12 plots each) in a panel of 89 bread wheat cultivars in the dry environment of the Karaganda region, Northern Kazakhstan**.

TABLE S2 **A list of the 19 Amplifluor SNP markers, references to the annotated contigs, corresponding BLAST results and sequences with SNP positions**.
