# Corrigendum: Expression Level of the *DREB2*-Type Gene, Identified with Amplifluor SNP Markers, Correlates with Performance, and Tolerance to Dehydration in Bread Wheat Cultivars from Northern Kazakhstan

**DOI:** 10.3389/fpls.2017.01571

**Published:** 2017-09-07

**Authors:** Yuri Shavrukov, Aibek Zhumalin, Dauren Serikbay, Makpal Botayeva, Ainur Otemisova, Aiman Absattarova, Grigoriy Sereda, Sergey Sereda, Vladimir Shvidchenko, Arysgul Turbekova, Satyvaldy Jatayev, Sergiy Lopato, Kathleen Soole, Peter Langridge

**Affiliations:** ^1^School of Agriculture, Food and Wine, Faculty of Sciences, University of Adelaide Urrbrae, SA, Australia; ^2^School of Biological Sciences, Flinders University Bedford Park, SA, Australia; ^3^Faculty of Agronomy, S. Seifullin Kazakh AgroTechnical University Astana, Kazakhstan; ^4^National Agricultural Research Education Centre Astana, Kazakhstan; ^5^Karaganda Research Institute of Plant Industry and Breeding Karaganda, Kazakhstan

**Keywords:** Amplifluor SNP, bread wheat, dehydration, *DREB2*, gene expression, genotyping, grain yield

In the original article, we neglected to acknowledge that data presented in the Supplementary Table S1 and Table 1 for grain yield were generated by Dr. Grigoriy Sereda in the framework of the project “Adaptawheat,” supervised by Dr. Yerlan Turuspekov, Institute of Plant Biology and Biotechnology, Almaty, Kazakhstan.

The authors apologize for this missing information and state that this does not change the scientific conclusions of the article in any way.

## Conflict of interest statement

The authors declare that the research was conducted in the absence of any commercial or financial relationships that could be construed as a potential conflict of interest.

